# Does natural selection explain the fine scale genetic structure at the nuclear exon *Glu-5′* in blue mussels from Kerguelen?

**DOI:** 10.1002/ece3.1421

**Published:** 2015-03-06

**Authors:** Karin Gérard, Charlotte Roby, Nicolas Bierne, Philippe Borsa, Jean-Pierre Féral, Anne Chenuil

**Affiliations:** 1 Institut Méditerranéen de Biodiversité et d'Ecologie marine et continentale - CNRS UMR 7263, Aix-Marseille UniversitéStation marine d'Endoume, 13007, Marseille, France; 2 Université Montpellier 2, Place Eugène Bataillon34095, Montpellier, France; 3 CNRS - Institut des Sciences de l'Evolution, UMR5554, Station Méditerranéenne de l'Environnement LittoralSète, France; 4 Institut de recherche pour le développement, UR227Montpellier, France; 5 CNRS UMR 6540 – DIMAR, Centre d'océanologie de MarseilleStation marine d'Endoume, 13007, Marseille, France

**Keywords:** AMOVA, environment, F-statistics, local adaptive differentiation, *mac-1*, neutral structure

## Abstract

The Kerguelen archipelago, isolated in the Southern Ocean, shelters a blue mussel *Mytilus* metapopulation far from any influence of continental populations or any known hybrid zone. The finely carved coast leads to a highly heterogeneous habitat. We investigated the impact of the environment on the genetic structure in those Kerguelen blue mussels by relating allele frequencies to habitat descriptors. A total sample comprising up to 2248 individuals from 35 locations was characterized using two nuclear markers, *mac-1* and *Glu-5′*, and a mitochondrial marker (*COI*). The frequency data from 9 allozyme loci in 9 of these locations were also reanalyzed. Two other nuclear markers (*EFbis* and *EFprem's*) were monomorphic. Compared to Northern Hemisphere populations, polymorphism in Kerguelen blue mussels was lower for all markers except for the exon *Glu-5′*. At *Glu-5′*, genetic differences were observed between samples from distinct regions (*F*_CT_ = 0.077), as well as within two regions, including between samples separated by <500 m. No significant differentiation was observed in the AMOVA analyses at the two other markers (*mac-1* and *COI*). Like *mac-1*, all allozyme loci genotyped in a previous publication, displayed lower differentiation (Jost's D) and *F*_ST_ values than *Glu-5′*. Power simulations and confidence intervals support that *Glu*-5′ displays significantly higher differentiation than the other loci (except a single allozyme for which confidence intervals overlap). AMOVA analyses revealed significant effects of the giant kelp *Macrocystis* and wave exposure on this marker. We discuss the influence of hydrological conditions on the genetic differentiation among regions. In marine organisms with high fecundity and high dispersal potential, gene flow tends to erase differentiation, but this study showed significant differentiation at very small distance. This may be explained by the particular hydrology and the carved coastline of the Kerguelen archipelago, together with spatially variable selection at *Glu*-5′.

## Introduction

In marine benthic organisms, a long planktonic larval stage generally allows gene flow between remote populations and consequently neutral genetic differentiation increases only slightly with geographical distance (Launey et al. 2002). Physical isolation (e.g., large distances, oceanic fronts, gyres) enhances genetic differences among populations. Differentiation may also arise locally through adaptation to localized environmental conditions (Maynard Smith 1966; Barton and Hewitt 1985). However, detecting adaptation through natural selection is difficult mainly because gene flow counters its effects at each generation (Kawecki and Ebert 2004; Sanford and Kelly 2011). Also, large variance in reproductive success (see Hedgecock's sweepstake reproduction hypothesis: Hedgecock 1994; Hedgecock and Pudovkin 2011) can generate transient chaotic patterns of genetic structure, known as ‘chaotic genetic patchiness’ (Johnson and Black 1984; Broquet et al. 2013) that sometimes resembles local adaptation. Differentiation between neighboring populations may be initiated by localized spatial heterogeneity in the environment such as hydrological characteristics (currents, exposition to wave action, salinity, temperature) or a complex topography (coastal shape, depth). Under conditions of fine-grained environmental heterogeneity, genetic differentiation at a selected locus may be higher between populations that differ environmentally even over short distances, than at other loci (Kawecki and Ebert 2004; Gagnaire et al. 2012).

**Figure 1:**
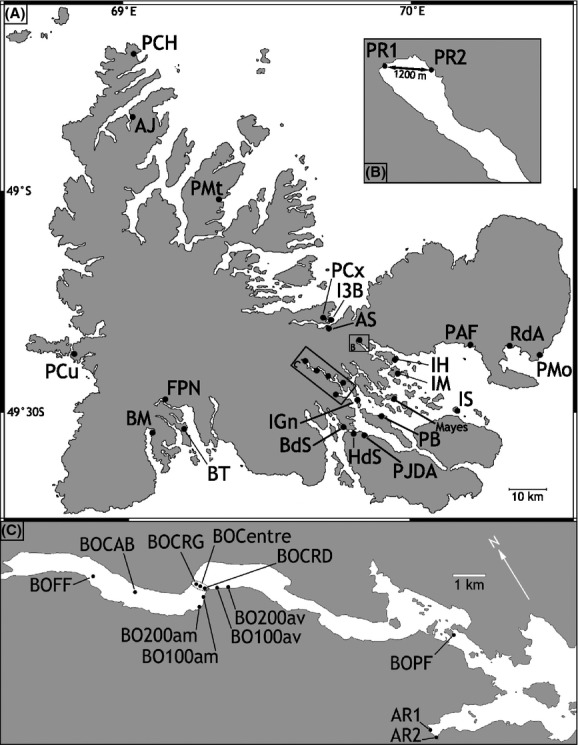
Sampling sites of Kerguelen blue mussels. Sampling details in Table1; (A) Main map. (B) Detail of locations for samples PR1 and PR2. (C) Enlarged map of Henri Bossière Fjord with locations of samples AR and BO. *Mayes* is the location of the sample ‘KER’ in Borsa et al. (2007).

The present work takes place in the Kerguelen archipelago, isolated in the southern Indian Ocean 4100 km southeast of South Africa and 4000 km west of Australia. The Kerguelen plateau is an obstacle to the eastward flow of the Antarctic Circumpolar Current and creates a large wake zone where water masses strongly mix (Park et al. 2008a,b). The cold superficial Antarctic Waters reach the west coast of the archipelago and separate into two parts drifting along south- and northward (Murail et al. 1977; Edgar 1987; Blain et al. 2001). The morphology of the Kerguelen Archipelago is the result of the volcanic activity combined with glacial erosion that led to a carved coast with protected bays and fjords, and a large enclosed bay with particular environmental conditions, the Gulf of Morbihan. In the very coastal perimeter, the salinity decreases drastically due to the important hydrographical network, and decreases even stronger in the shallow waters of the gulf, or deep inside the bays and fjords like at the *Fjord des Portes Noires* and the *Fjord Henri Bossière* (Fig.1) (Arnaud 1974; Murail et al. 1977). This archipelago is also characterized by a high level of endemism (Briggs 1966; McDowall 1968; Poulin and Féral 1995; Hennion and Walton 1997; Brandt et al. 1999; Frenot et al. 2001; Emerson 2002). Given the discrete geographical nature (Emerson 2002) and spatial heterogeneity of the environment, Kerguelen Islands seem particularly suited to investigate the association of the environment and the population differentiation in a marine species with a long planktonic larval stage (thereby a putatively high dispersal potential), such as smooth-shelled *Mytilus* (L.) mussels.

The blue mussels from Kerguelen are mainly distributed in the intertidal zone from 0 to 2 m depth (Arnaud 1971, 1974), where the environmental conditions are the most variable. Kerguelen blue mussels have been described as *M. desolationis* (Lamy, 1936). However, their current taxonomic status, determined from morphology, allozymes, and nuclear and mitochondrial DNA sequences, is *M. edulis platensis,* the Southern-Hemisphere subspecies of *M. edulis* L. (McDonald et al. 1991; Borsa et al. 2012). Genetic differentiation between smooth-shelled *Mytilus* spp. mussels (*M. edulis* L., *M. galloprovincialis* Lmk., *M. trossulus* Gould) and the phylogeography of these species has been studied extensively (e.g., McDonald et al. 1991; Sanjuan et al. 1997; Daguin 2000; Daguin and Borsa 2000; Hilbish et al. 2000; Gérard et al. 2008), with particular focus on areas of hybridization (Skibinski et al. 1978; Skibinski 1983; Väinölä and Hvilsom 1991; Viard et al. 1994; Gardner 1996; Bierne et al. 2002a,b, 2003). The most famous example of genetic differentiation linked to the environment in blue mussels is the gradient at the *lap* locus correlated with salinity gradient along the eastern coast of North America (Koehn 1978). The physiological and selective roles of the *lap* locus have been highlighted (Hilbish et al. 1982; Hilbish and Koehn 1985a,b) and further studied in blue mussels from Kerguelen (Blot et al. 1989) and New Zealand (Gardner and Kathiravetpillai 1997; Gardner and Palmer 1998). In Kerguelen blue mussels, genetic differences between populations were apparent at three (*lap, pgm*, *pgd*) allozyme loci (Blot et al. 1989), and the structure reported to be related to salinity, wave exposure and, to a lesser extent, to the maximum shell length (as a proxy of fitness). However, no statistical analyses were conducted to support this conclusion. Theoretically, genetic differentiation may be due to physical barriers to gene flow but also to local adaptation under selective constraints (Williams 1966; Kawecki and Ebert 2004; Perrin et al. 2004). In cases of barriers to gene flow, the differentiation will affect a majority of loci, whereas in cases of local adaptation only a few loci are concerned.

To determine whether the genetic polymorphism of the blue mussel population of Kerguelen is driven by neutral and/or adaptive forces, we (i) investigated the influence of the water circulation around Kerguelen, first on the total genetic structure and second within differentiated groups and (ii) tested the influence of the habitat type at a smaller scale. To fill in these objectives, we used two nuclear markers polymorphic in Kerguelen blue mussels: *Glu-5′* (Inoue et al. 1995; Rawson et al. 1996) and *mac-1* (Ohresser et al. 1997), and we also considered the sequence polymorphism at the mitochondrial DNA locus *COI* (Gérard et al. 2008). We tested the polymorphism at *EFbis* (Bierne et al. 2002a) and *EFprem's* (this study), two introns of the elongation factor 1 alpha gene, which are physically linked. We collected blue mussel samples from all around the Kerguelen Archipelago, from contrasted habitats roughly described by five qualitative environmental variables. At a finer grid, a dense network of sites in the complex of islands of the Gulf of Morbihan was sampled to explore the distribution of the allele frequencies, taking into account the environmental changes over short distances.

## Material and Methods

### Sampling

Blue mussel samples were collected between 1999 and 2003 from 35 sites in the Kerguelen Archipelago (Fig.1). Sample size ranged from 27 to 130 individuals (Table1). Five samples came from the north coast (PCh, PMt, PCx, I3B, AJ), two from the east coast (PMo, RdA), twenty-two from the Gulf of Morbihan (PAF, PR1, PR2, IH, IM, IGn, PF, BOCRD, BOCentre, BOCRG, BO100am, BO200am, BO100av, BO200av, BOCAB, BOFF, Ar1, Ar2, HdS, PJDA, PB, IS), Four from the south coast (BdS, BM, FPN, BM) and one from the west coast (PCu). Pieces of mantle tissue were preserved in 95% ethanol. DNA was extracted using Chelex100© chelating resin (Walsh et al. 1991).

**Table 1:** Sample location of Kerguelen blue mussels

Site	Abbreviation	Region	GPS coordinates
*Port Christmas*	PCh	North	48°40′58″; 69°02′12″
*Anse du Jardin*	AJ	North	48°50′14″; 69°00′43″
*Port Matha*	PMt	North	48°55′59″; 69°02′35″
*Port Couvreux*	PCx	North	49°17′07″; 69°41′50″
*Ilot des Trois Bergers*	I3B	North	49°17′17″; 69°42′23″
*Anse Sablonneuse*	AS	North	49°18′53″; 69°42′30″
*Pointe Morne*	PMo	East	49°22′47″; 70°26′36″
*Rivière des Albatros*	RdA	East	49°21′08″; 70°22′47″
*Port-aux-Français*	PAF	Gulf of M.	49°21′08″; 70°13′07″
*Port Raymond* (deep bay)	PR1	Gulf of M.	49°20′19″; 69°48′20″
*Port Raymond* (Hut)	PR2	Gulf of M.	49°20′27″; 69°49′22″
*Ile haute*	IH	Gulf of M.	49°23′13″; 69°56′20″
*Ile aux Moules*	IM	Gulf of M.	49°25′04″; 69°56′54″
*Ile Guillou* (north)	IGn	Gulf of M.	49°28′10″; 69°48′23″
*Portes de Fer*	PF	Gulf of M.	49°26′34″; 69°45′
*Henri Bossière* Fjord, 200 m downstream from mb	BO200av	Gulf of M.	See Fig.1
*Henri Bossière* Fjord, 100 m downstream from mb	BO100av	Gulf of M.	See Fig.1
*Henri Bossière* Fjord, eastern part of mb	BOCRD	Gulf of M.	See Fig.1
*Henri Bossière* Fjord, center of mb	BOCentre	Gulf of M.	49°24′28″; 69°40′06″
*Henri Bossière* Fjord, western part of mb	BOCRG	Gulf of M.	See Fig.1
*Henri Bossière* Fjord, 100 m upstream from mb	BO100am	Gulf of M.	See Fig.1
*Henri Bossière* Fjord, 200 m upstream from mb	BO200am	Gulf of M.	See Fig.1
*Henri Bossière* Fjord, front of hut	BOCAB	Gulf of M.	49°24′07″; 69°38′37″
*Henri Bossière* Fjord, deep part	BOFF	Gulf of M.	49°23′36″; 69°37′48″
*Armor*	Ar1	Gulf of M.	49°27′50″; 69°43′40″
*Armor*	Ar2	Gulf of M.	49°27′50″; 69°43′40″
*Halage des Swains*	HdS	Gulf of M.	49°32′57″; 69°48′05″
*Port Jeanne d'Arc*	PJDA	Gulf of M.	49°33′07″; 69°49′26″
*Port Bizet*	PB	Gulf of M.	49°31′02″; 69°54′13.″
*Ile Suhm*	IS	Gulf of M.	49°30′18″; 70°09′38″
*Baie des Swains*	BdS	South	49°33′02″; 69°46′23″
*Baie de la Table*	BT	South	49°31′; 69°11′15″
*Fjord des Portes Noires*	FPN	South	49°29′; 69°06′06″
*Baie de la Mouche*	BM	South	49°32′56″; 69°05′10″
*Port Curieuse*	PCu	West	49°21′41″; 68°48′35″

mb, mussel bed.

### Molecular markers

**Figure 2:**
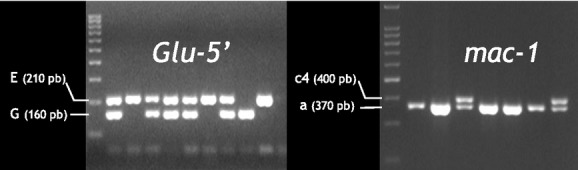
Kerguelen blue mussels. Individual phenotypes scored on agarose gels at nuclear loci. The left lane is a 100-bp DNA ladder.

The locus *Glu-5′* (Inoue et al. 1995; Rawson et al. 1996) is located at the 5′ extremity of exon *Glu* coding for an adhesive foot protein (Waite 1992). This locus contains an insertion/deletion (indel) zone, whose amplification reveals three alleles: (*T*, *E* and *G*) that, respectively distinguish *M. trossulus*, *M. edulis,* and *M. galloprovincialis* in the Northern Hemisphere (Borsa et al. 1999). The locus *mac-1* is the first intron of the *Mytilus* actin protein (Ohresser et al. 1997). Among the 49 size-alleles described in the entire range of *Mytilus* spp., 22 alleles occur in the Southern Hemisphere, 8 of which have not yet been sampled in the Northern Hemisphere (Daguin and Borsa 2000). The four alleles encountered in the Kerguelen blue mussel population in Kerguelen are all shared with Northern-Hemisphere populations (Daguin and Borsa 2000; Borsa et al. 2007). The polymorphism at locus *EFbis* has been tested in one location in Kerguelen (Mayes Island in the Gulf of Morbihan) and was low, with two alleles detected (160: frequency 0.01; 161: 0.99) (Daguin 2000). We scored locus *EFBis* in other samples from around the archipelago. A new EPIC marker (*EFprem's)* in the second intron of EF1*α* was also scored. Both *EFBis* and *EFprem's* loci showed sample monomorphism (see Results). Table S1 summarizes primer names, sequences, and annealing temperatures required for the amplification of nuclear and mitochondrial DNA loci. The genotypes at *Glu-5′* and *mac-1* were determined from fragment-length variation on, respectively, 2 and 3% agarose gels. The amplification of the *Glu-5′* exon by primers *Me-15* and *Me-17* produced 210-bp (allele *E*) and 160-bp (allele *G*) fragments, typical of, respectively, *M. edulis* and *M. galloprovincialis* from the Northern Hemisphere (Fig.2A). At locus *mac-1*, fragments of 400 and 370 bp were revealed. According to Daguin (2000) and Borsa et al. (2007), the 400-bp fragment at locus *mac-1* corresponds to allele *c4*, whereas the 370-bp fragment corresponds to either allele *a2* or *a3*, which differ from one another by one base pair and cannot be distinguished on agarose gels (Fig.2B). Consequently, the 370-bp fragment is here noted ‘*a’*. The denomination of *COI* haplotypes (KERF1 to KERF16) follows Gérard et al. (2008).

### Genetic analysis

Heterozygosity was estimated by Nei's (1978) nonbiased heterozygosity index (*H*_n.b_). *F*_IS_ and *F*_ST_ values were estimated according to Weir and Cockerham (1984) using the FSTAT procedure in the program GENETIX 4.02 (Belkhir et al. 2000). The significance of *F*_IS_ (and respectively *F*_ST_) values was assessed after 5000 permutations of alleles (resp. individuals) within (resp. between) samples, thus obtaining the distribution of *F*_IS_ (resp. *F*_ST_) pseudovalues under the null hypothesis of panmixia (resp. a nonstructured population). The probability (*P*) values associated to *F*_IS_ or *F*_ST_ estimates was the proportion of pseudovalues generated by 5000 random permutations larger than, or equal to the observed value. Mantel tests were used to assess the correlation of pairwise *F*_ST_ values and geographical distances (by coastal line) between samples computing the association statistics Z (Mantel 1967). The *P*-value of Z was the proportion of pseudovalues generated by permutations under the null hypothesis of independence of genetic and geographical distances, larger than, or equal to the observed value of Z. Mantel tests and permutations were computed by the program GENETIX 4.02.

The false discovery rate correction for multiple comparisons was used to adjust levels of statistical significance (Benjamini and Hochberg 1995).

Analyses of molecular variance (AMOVA; Excoffier et al. 1992) were carried out on *Glu-5′* and *mac-1* genotype data, as well as on *COI* sequences (conventional *F*_ST_ based on frequencies) for comparative purposes using ARLEQUIN 3.0 (Excoffier et al. 2005). Following the results of pairwise *F*_ST_ (see Results), we defined and tested a geographical structure of three groups based on the origin of the samples (North + East, Gulf, South + West; Table1). In the AMOVA grouping, the unique sample from the west coast (PCu) was lumped with the South group, from which it was not differentiated (see pairwise *F*_ST_ results; Table4). The exact test of sample differentiation (Raymond and Rousset 1995) was run (with 20,000 Markov chain, 1000 dememorization steps) using this same software based on mitochondrial haplotype frequency in the three groups. So-called neutrality tests (Fu's Fs and Tajima's D) were run to check whether the double hypothesis of demographic stability and selective neutrality of the *COI* marker could be rejected.

We reanalyzed gene frequency data of nine allozyme loci published in Blot (1989) and Blot et al. (1989). Genotypes were not available, but we could compute *F*_ST_ from gene frequency data using the relationship *F*_ST_ = 1 – Ho/He, the exact tests of differentiation based on Jost's differentiations (see below and Table2), and the contingency tables of the numbers of each allele in each population (Table S2).

**Table 2:** Kerguelen blue mussels. Jost's D differentiation estimates and its confidence interval (CI) calculated using a set seven populations common to the allozyme study of Blot (1989) and Blot et al. (1989) and present study. Populations were PMt and PCx from the North region, PAF, IS, HdS, and BOS from the Gulf of Morbihan (all samples within the Bossière Fjord were pooled), and BT (*Glu-5′* and *mac-1*) or “Larose” (allozymes) from the South region. Bold values have confidence intervals that do not include zero

Locus	D	CI
*Glu-5′*	**0.049**	**0.023–0.075**
*Mac-1*	0.002	0.000–0.008
*LAP1*	0.014	0.000–0.032
*LAP2*	0.011	0.000–0.025
*PGD*	**0.032**	**0.017–0.047**
*PGI*	0.009	0.000–0.027
*PGM*	0.007	0.000–0.018
*GOT1*	0.000	0.000–0.000
*GOT2*	0.001	0.000–0.002
*MDH1*	0.001	0.000–0.003
*MDH2*	0.001	0.000–0.003

Two approaches were used to compare differentiation levels among markers or data sets with contrasted samples sizes and polymorphism levels. (i) Jost's differentiation parameters (D) and confidence intervals were computed using the program SPADE (Jost 2008; Chao and Shen 2010). This was carried out for the common subset of seven populations which were analyzed in the present study (for *Glu-5′* and *mac-1*), in Blot (1989) and Blot et al. (1989) for nine allozyme markers. The interest in this approach is that Jost's D is much less affected by polymorphism level than *F*_ST_ and provides confidence intervals. (ii) The POWSIM application (Ryman and Palm 2006) was used to compare results among markers which did not display similar sample sizes and allele frequency distributions. This application uses simulated data sets corresponding to a model of diverging populations (no migration): a given *F*_ST_ level is chosen by the user, by selecting an appropriate pair of values for effective size and divergence time (in number of generations). We thus checked whether the small sample sizes or the reduced polymorphism for the *COI* and *mac-1* data sets, respectively, relative to those of *Glu-5′* may affect our results in the finding of significant differentiation among regions. The simulations were run at the differentiation level found with the *Glu-5′* genotypic data (we used a value similar to both the overall *F*_ST_ among all populations and the global *F*_CT_ among regions), using the global frequency distributions of *COI* haplotypes and *mac-1* alleles and their respective sample sizes in the three groups of populations (North + East, Gulf, South + West). The output of the program provides the proportion of cases in which significant differentiation is found.

A median-joining parsimony network (Bandelt et al. 1999) of *COI* haplotypes was built using NETWORK 4.1.0.7 (available at www.fluxus-technology.com/).

### Environmental factors

The habitat at each sampling site was described by five qualitative variables: (i) Substrate (rock, blocks, gravels, or sand); (ii) Wave Exposure (sheltered or exposed); (iii) Slope (flat, steep or hangover); (iv) Salinity (oceanic or influenced by freshwater); (v) *Macrocystis* (presence or absence). The Region (North, South, Gulf, and West) was also considered as a factor in the following statistical analyses. Correlation among environmental factors and frequency of allele *G* was assessed by pairwise Spearman's *ρ* values (Spearman 1904).

We also used AMOVAs on *Glu-5′* and *mac-1* genotypic data and *COI* haplotype data, within each geographical region. For each environmental factor, we grouped samples according to the modality of the variable in order to test the effects of environment within regions (*F*_CT_, Va), within groups independently of the effects of population differentiation between regions. The AMOVA, although it is restricted to investigate nested factors, has two important advantages over parametric analyses (ANCOVAs were performed using the proportion of the *G* allele at *Glu-5′* as the variable to explain, but not shown): (i) it does not rely upon statistical conditions on the distribution of the data, as the *P*-value is assessed via permutations (Excoffier et al. 2005) and (ii) it takes into account the statistically important information of the number of individuals in each population.

## Results

### Polymorphism

The number of individuals characterized was 2248, 1511, and 83 for *Glu-5′*, *mac-1*, and *COI* (Table3), respectively, and 20 for each *EFbis* and *EFprem's*.

**Table 3:** Allelic frequencies at loci *Glu-5′*, *mac-1,* and haplotype composition at locus *COI* for each sample

Region	Sample	Locus										Environmental factors				
		*Glu-5′*				*mac-1*				*COI*		Substrate	Slope	Exposure	Salinity	*Macro-cystis*
		*N*	Frequency of allele *G*	*H*_n.b._	*f*	*N*	Frequency of allele *a*	*H*_n.b._	*f*	*N*	Haplotype					
North	PCh	51	0.26	0.393	−0.149 ns	49	0.9	0.185	0.346 ns	4	F1, F3 (3)	R	H	Sh	OW	P
North	AJ	106	0.41	0.485	−0.091 ns	49	0.92	0.152	−0.079 ns	–		G	St	Sh	OW	P
North	PMt	50	0.24	0.369	0.133 ns	48	0.88	0.221	−0.134 ns	2	F1, F5	G	St	Sh	OW	P
North	PCx	109	0.2	0.324	0.150 ns	48	0.96	0.081	−0.033 ns	4	F4, F5 (2), F12	B	H	E	OW	P
North	I3B	50	0.17	0.285	0.230 ns	49	0.94	0.116	0.303 ns	4	F4 (2), F5, F12	R	H	E	OW	P
North	AS	54	0.21	0.338	−0.151 ns	50	0.94	0.114	0.304 ns	–		G	F	Sh	OW	A
North	PMo	50	0.24	0.369	−0.086 ns	50	0.92	0.149	−0.078 ns	2	F2, F16	B	St	E	OW	P
North	RdA	50	0.09	0.166	0.155 ns	50	0.83	0.285	0.233 ns	3	F1 (3)	S	F	Sh	LSW	A
Gulf of M.	PAF	50	0.45	0.500	0.161 ns	48	0.93	0.137	−0.069 ns	2	F2, F4	B	St	E	OW	P
Gulf of M.	PR1	50	0.52	0.504	0.129 ns	49	0.93	0.134	−0.067 ns	4	F4, F5 (4)	S	F	Sh	LSW	A
Gulf of M.	PR2	50	0.45	0.500	0.000 ns	49	0.81	0.316	0.031 ns	–		G	F	Sh	LSW	P
Gulf of M.	IH	116	0.44	0.495	−0.186*	79	0.95	0.097	−0.047 ns	5	F1, F5, F13, F14, F15	R	H	E	OW	P
Gulf of M.	IM	118	0.44	0.494	0.091 ns	78	0.94	0.121	−0.062 ns	1	F5	R	St	E	OW	P
Gulf of M.	PJDA	115	0.48	0.501	0.168 ns	79	0.94	0.119	−0.062 ns	–		B	St	E	OW	P
Gulf of M.	HdS	71	0.48	0.503	0.048 ns	69	0.95	0.097	−0.046 ns	3	F3, F5 (2)	B	F	Sh	OW	A
Gulf of M.	PB	127	0.46	0.500	0.086 ns	79	0.91	0.163	−0.091 ns	3	F1, F2 (2)	R	H	E	OW	P
Gulf of M.	IGn	77	0.6	0.484	0.035 ns	61	0.93	0.124	0.207 ns	3	F4, F5 (2)	B	F	Sh	OW	A
Gulf of M.	Ar1	50	0.72	0.407	−0.081 ns	48	0.93	0.137	−0.069 ns	2	F1, F2	R	F	Sh	LSW	A
Gulf of M.	Ar2	28	0.54	0.506	0.013 ns	30	0.87	0.235	0.151 ns	–		R	H	Sh	OW	A
Gulf of M.	BO200av	19	0.47	0.512	−0.029 ns	–	–	–	–	–		G	F	Sh	LSW	A
Gulf of M.	BO100av	49	0.47	0.503	0.109 ns	–	–	–	–	–		G	F	Sh	LSW	A
Gulf of M.	BOCRD	50	0.55	0.500	−0.243 ns	–	–	–	–	–		S	F	Sh	LSW	A
Gulf of M.	BOCentre	50	0.6	0.485	0.093 ns	49	0.94	0.116	−0.055 ns	5	F2, F3, F5, F9, F10	S	F	Sh	LSW	A
Gulf of M.	BOCRG	50	0.54	0.502	0.044 ns	–	–	–	–	–		S	F	Sh	LSW	A
Gulf of M.	BO100am	50	0.57	0.495	0.072 ns	–	–	–	–	–		G	F	Sh	LSW	A
Gulf of M.	BO200am	49	0.51	0.505	0.112 ns	–	–	–	–	–		G	F	Sh	LSW	A
Gulf of M.	BoFF	50	0.46	0.502	−0.198 ns	49	0.95	0.098	−0.044 ns	3	F2, F4, F5	S	F	Sh	LSW	A
Gulf of M.	BoCAB	49	0.45	0.500	−0.145 ns	50	0.96	0.078	0.494 ns	–		B	F	Sh	LSW	A
Gulf of M.	PF	27	0.43	0.498	0.335 ns	26	0.92	0.145	−0.065 ns	2	F1, F4	R	H	E	LSW	A
Gulf of M.	IS	40	0.39	0.481	0.222 ns	29	0.88	0.216	−0.122 ns	4	F2, F3, F4, F5	B	St	E	OW	P
South	BdS	69	0.37	0.469	0.167 ns	75	0.88	0.212	−0.129 ns	3	F1, F4, F5	B	F	Sh	OW	P
South	BT	50	0.32	0.440	−0.093 ns	30	0.85	0.259	−0.150 ns	3	F5 (2), F11	R	H	E	OW	P
South	FPN	130	0.37	0.470	0.001 ns	50	0.94	0.114	−0.054 ns	3	F1, F4, F12	S	F	Sh	LSW	P
South	BM	108	0.28	0.403	0.035 ns	56	0.92	0.149	−0.079 ns	2	F5, F8	R	H	E	OW	P
West	PCu	36	0.4	0.488	−0.083 ns	35	0.96	0.083	−0.031 ns	16	F1 (3), F2 (3), F3 (2), F4 (1), F5 (3), F6, F7, F12 (2)	R	H	E	OW	P

*N*, sample size; *H*_n.b_., nonbiased estimate of genetic diversity; *f*, Weir and Cockerham's (1984) estimate of Wright's *F*_IS_; *, significant values at the level of 5%; ns, non-significant. Substrate: rock (R), blocks (B), gravels (G), sand (S). Slope: flat (F), steep (St), hangover (H). Wave exposure: sheltered (Sh), exposed (E). Salinity: oceanic water (OW), low-salinity water (LSW). *Macrocystis*: presence (P)/absence (A).

**Figure 3:**
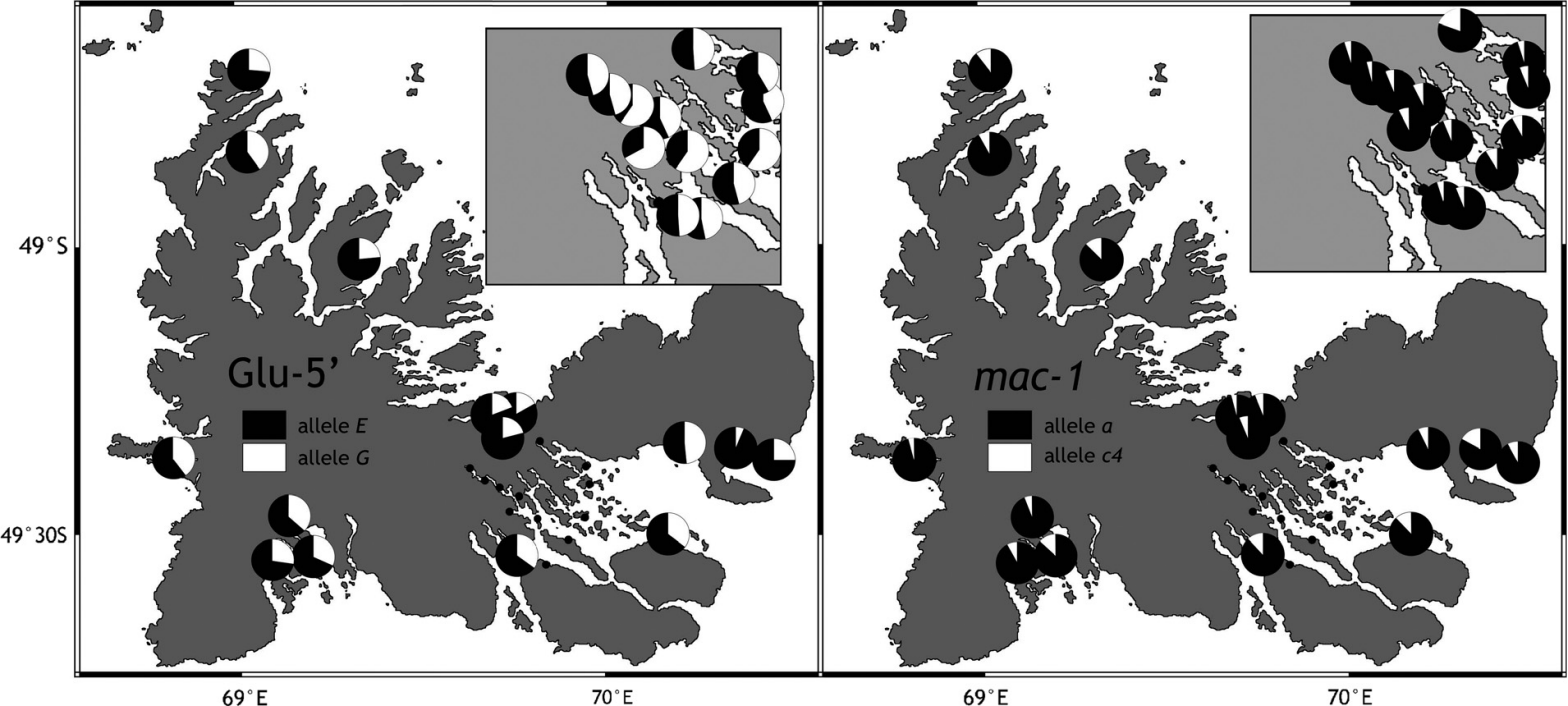
Kerguelen blue mussels. Geographic distribution of allele frequencies at the nuclear loci *Glu-5′* and *mac-1*. Insets: samples from the fjords and islands in the western part of the Gulf of Morbihan.

**Figure 4:**
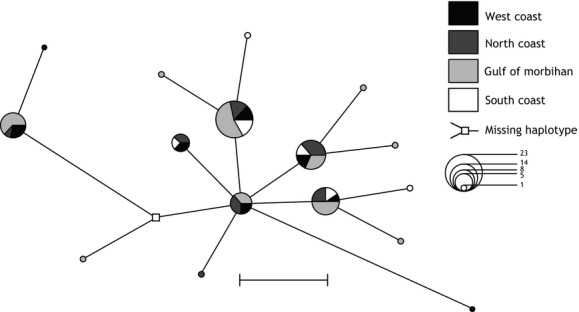
Median-joining parsimony network of *COI* haplotypes sampled in Kerguelen blue mussels. Scale bar represents one mutational step.

We detected no polymorphism at loci *EFbis* and *EFprem's* scored on agarose gels. At locus *Glu-5′*, *G* and *E* allelic frequencies were of 41.6 and 58.4% in the total sample. In the Gulf of Morbihan, the *G* allele occurred in higher frequencies, sometimes over 50% (samples PR1, IGn, Ar1, Ar2, BoCRD, Bo100am, Bo200am, BoCentre; Table3, Fig.3). At *mac-1*, the allele *a* had a frequency of 91.9% in the total sample. Allele *c4* had the lowest frequency in all samples (from 4 to 19.7%; Table3, Fig.3). Average *H*_n.b._ values were of 0.453 and 0.152, respectively, for *Glu-5′* and *mac-1*. *F*_IS_ values at the two nuclear loci were generally nonsignificantly different from 0 (Table3), except for the sample IH (northern part of the Gulf) which shows a heterozygote excess (*F*_IS_ = −0. 186; *P* = 0.0362), but this significance level did not pass, by far, the correction for multiple tests. At locus *COI*, 16 haplotypes were found, haplotype diversity was about *H*_n.b._ = 0.86 within region (it was not computed within population due to a small sample sizes). Neutrality tests (Tajima's D and Fu's Fs) within each region were nonsignificant, and the haplotype network appeared balanced (Fig.4).

### Differentiation among populations

Overall *F*_ST_ values at both nuclear DNA loci were significant (*Glu-5′*: *F*_ST_ = 0.0627 ± 0.0176, *P* ≤ 0.0001; *mac-1*: *F*_ST_ = 0.00945 ± 0.00592; *P* ≤ 0.004) establishing the presence of significant genetic structure for these loci in Kerguelen. By contrast, the exact test of global differentiation at the *COI* locus did not appear significant (*P* = 0.59856 ± 0.08481), and the single non-negative estimate of *F*_ST_ value among regions (pooling individuals from different populations) was 0.007 (nonsignificant).

At *Glu-5′*, pairwise *F*_ST_ values revealed a differentiation between samples depending on the region to which they belong. Three groups of genetically differentiated samples may be identified (Table1 and 3): (i) the northern group presented the highest frequencies of the *E* allele, (ii) the Gulf group presented the highest frequencies of the *G* allele; (iii) the southern group was intermediate. The unique sample from the west coast (PCu) was less differentiated from the southern group than from the northern group and the Gulf. At locus *Glu-5′* after the FDR correction, only *P*-values lower than 0.021 subsisted, mainly those concerning pairwise *F*_ST_ between samples from the north coast and the Gulf of Morbihan. At locus *mac-1*, fewer pairwise *F*_ST_ values were significant (Table4). However, the samples RdA (East Coast) and PR2 (Gulf) are significantly differentiated from the majority of the other samples, due to the high frequency of allele *c4* (17 and 19.4%, respectively). Only PR2 remained differentiated from the remaining samples after FDR correction.

**Table 4:** Kerguelen blue mussels. Pairwise *F*_ST_ (Weir and Cockerham 1984) values at *Glu-5′* (above diagonal) and *mac-1* (below diagonal) loci

Region	Sample	North								Gulf of Morbihan							
		PCh	AJ	PMt	PCx	I3B	AS	PMo	RdA	PAF	PR1	PR2	IH	IM	PJDA	HdS	PB
North	PCh	–	0.036*	0	0.004	0.016	0	0	**0.091**	0.063**	0.119**	**0.063**	0.057**	**0.053**	**0.082**	**0.084**	0.072**
	AJ	0	–	0.051**	**0.09**	**0.111**	**0.073**	**0.052**	**0.202**	0	0.019*	0	0	0	0.006	0.005	0.003
	PMt	0	0.001	–	0	0.003	0	0	0.068*	0.082**	**0.144**	0.083**	0.075**	**0.071**	0.103**	0.106**	**0.092**
	PCx	0.014	0.004	0.035*	–	0	0	0	0.036*	**0.133**	**0.205**	**0.134**	**0.117**	**0.114**	**0.152**	**0.158**	**0.138**
	I3B	0	0	0.013	0	–	0	0.004	0.016	**0.158**	**0.23**	**0.159**	**0.139**	**0.135**	**0.173**	**0.181**	**0.159**
	AS	0	0	0.015	0	0	–	0	0.048*	**0.111**	**0.177**	0.112**	**0.099**	**0.095**	**0.123**	**0.135**	**0.118**
	PMo	0	0	0.002	0.003	0	0	–	0.07**	0.083**	**0.145**	0.084**	**0.075**	**0.071**	**0.103**	**0.106**	**0.092**
	RdA	0.007	0.024	0	0.071**	0.044*	0.046*	0.025*	–	**0.274**	**0.351**	**0.275**	**0.232**	**0.228**	**0.269**	**0.289**	**0.253**
Gulf of M.	PAF	0	0	0.006	0	0	0	0	0.032*	–	0	0	0	0	0	0	0
	PR1	0	0	0.007	0	0	0	0	0.033*	0	–	0	0.006	0.006	0	0	0
	PR2	0.021	0.042*	0.008	0.096**	0.065**	0.068**	0.044*	0	0.051*	0.053*	–	0	0	0	0	0
	IH	0.010	0	0.030*	0	0	0	0	0.069**	0	0	**0.095**	–	0	0	0	0
	IM	0	0	0.01554	0	0	0	0	0.049**	0	0	0.072**	0	–	0	0	0
	PJDA	0.001	0	0.01636	0	0	0	0	0.050**	0	0	0.074**	0	0	–	0	0
	HdS	0.009	0	0.023*	0	0	0	0	0.066**	0	0	0.091**	0	0	0	–	0
	PB	0	0	0	0.009	0	0	0	0.022	0	0	0.04**	0.005	0	0	0.005	–
	IGn	0	0	0.012	0	0	0	0	0.042*	0	0	0.064	0	0	0	0	0
	Ar1	0	0	0.006	0	0	0	0	0.032*	0	0	0.051*	0	0	0	0	0
	Ar2	0	0.001	0	0.043*	0.016	0.017	0.002	0	0.007	0.008	0	0.037*	0.019	0.020	0.035*	0
	BOCRD	–	–	–	–	–	–	–	–	–	–	–	–	–	–	–	–
	BOCentre	0	0	0.015	0	0	0	0	0.045*	0	0	0.066**	0	0	0	0	0
	BO100av	–	–	–	–	–	–	–	–	–	–	–	–	–	–	–	–
	BO200av	–	–	–	–	–	–	–	–	–	–	–	–	–	–	–	–
	BO100am	–	–	–	–	–	–	–	–	–	–	–	–	–	–	–	–
	BO200am	–	–	–	–	–	–	–	–	–	–	–	–	–	–	–	–
	BOCRG	–	–	–	–	–	–	–	–	–	–	–	–	–	–	–	–
	BOFF	0.006	0	0.025*	0	0	0	0	0.058*	0	0	0.081**	0	0	0	0	0.003
	BOCab	0.015	0.004	0.037*	0	0	0	0.003	0.074*	0	0	0.099**	0	0	0	0	0.01
	PF	0	0	0.012	0	0	0	0	0.037	0	0	0.057*	0	0	0	0	0
	IS	0	0	0	0.034	0.008	0.009	0	0	0.001	0.002	0.006	0.027	0.011	0.012	0.025	0
South	BdS	0	0	0	0.025*	0.008	0.009	0	0.005	0.002	0.002	0.018	0.020*	0.01	0.01	0.012*	0
	BT	0	0.012	0	0.063**	0.031	0.033	0.014	0	0.020	0.022	0	0.057**	0.035*	0.04*	0.054**	0.01
	FPN	0	0	0.016	0	0	0	0	0.047*	0	0	0.069**	0	0	0	0	0
	BM	0	0	0.002	0.004	0	0	0	0.027*	0	0	0.045*	0	0	0	0	0
West	PCu	0.009	0.001	0.029	0	0	0	0	0.061*	0	0	0.084**	0	0	0	0	0.006

IGn	Ar1	Ar2	CRD	Centre	100av	200av	100am	200am	CRG	FF	Cab	PF	IS	South				West
														BdS	BT	FPN	BM	PCu
**0.193**	**0.338**	0.136**	**0.149**	**0.198**	0.077**	0.08*	0.167**	0.111**	0.138**	0.072**	0.063**	0.044*	0.023	0.016	0	0.019*	0	0.033*
**0.066**	**0.172**	0.024	0.035**	**0.066**	0.001	0	0.0461**	0.015	0.029*	0	0	0	0	0	0.009	0	0.031**	0
**0.220**	**0.369**	0.163**	**0.175**	**0.226**	0.099**	0.103*	**0.194**	**0.135**	**0.164**	0.092**	0.083*	0.062*	0.037*	0.03*	0.006	0.032*	0	0.049*
**0.283**	**0.432**	**0.229**	**0.238**	**0.293**	**0.153**	**0.162**	**0.26**	**0.198**	**0.227**	**0.144**	**0.133**	0.112**	0.078	**0.063**	0.030*	**0.064**	0.011	0.092**
**0.306**	**0.463**	**0.26**	**0.264**	**0.319**	**0.178**	**0.197**	**0.285**	**0.22**	**0.252**	**0.17**	**0.159**	**0.140**	0.102**	0.084**	0.049*	**0.084**	0.023	0.118**
**0.254**	**0.406**	**0.201**	**0.21**	**0.263**	**0.129**	0.139**	**0.23**	**0.168**	**0.198**	**0.122**	**0.112**	0.092**	0.061**	0.048**	0.021*	0.050**	0.004	0.074**
**0.221**	**0.369**	**0.164**	**0.176**	**0.227**	0.1**	0.105**	**0.195**	**0.136**	**0.165**	**0.093**	0.084*	0.064*	0.038*	0.029*	0.007	0.032**	0	0.05*
**0.415**	**0.579**	**0.403**	**0.386**	**0.441**	**0.3**	**0.354**	**0.407**	**0.341**	**0.373**	**0.287**	**0.275**	**0.276**	**0.22**	**0.179**	**0.142**	**0.167**	**0.091**	**0.239**
0.034*	0.13**	0	0.01	0.033	0	0	0.017	0	0.005	0	0	0	0	0.003	0.025	0.005	0.057**	0
0.003	0.072**	0	0	0.002	0	0	0	0	0	0	0	0	0.022	0.035*	**0.069**	0.036*	**0.113**	0.015
0.035*	**0.131**	0.001	0.011	0.034*	0	0	0.018	0	0.006	0	0	0	0	0.004	0.026*	0.005	0.057**	0
**0.044**	**0.14**	0.01	0.02*	0.044**	0	0	0.027*	0.003	0.014	0	0	0	0	0.005	0.023*	0.005	**0.051**	0
**0.045**	**0.141**	0.008	0.02	0.045**	0	0	0.028*	0.003	0.014	0	0	0	0	0.003	0.021*	0.004	**0.049**	0
0.022*	**0.104**	0	0.003	0.021	0	0	0.009	0	0	0	0	0	0.007	0.017	0.042**	0.018*	**0.077**	0.001
0.021	**0.105**	0	0.002	0.020*	0	0	0.007	0	0	0	0	0	0.006	0.016	0.043*	0.017*	**0.079**	0.001
0.023*	**0.115**	0	0.008	0.029*	0	0	0.014	0	0.004	0	0	0	0.003	0.012	0.035**	0.013*	**0.067**	0
–	0.024*	0	0	0	0.024	0.014	0	0.006	0	0.03*	0.036*	0.044*	**0.075**	**0.092**	**0.136**	**0.091**	**0.186**	0.064**
0	–	0.06**	0.05*	0.022	**0.113**	0.109**	0.038	0.079**	0.06**	**0.123**	**0.133**	**0.153**	**0.193**	**0.211**	**0.27**	**0.206**	**0.323**	**0.179**
0.014	0.007	–	0	0	0	0	0	0	0	0	0.002	0.003	0.027	0.042*	0.080**	0.043*	0.127**	0.02
–	–	–	–	0	0.003	0	0	0	0	0.008	0.012	0.017	0.041*	0.056**	**0.095**	**0.056**	**0.141**	0.033*
0	0	0.018		–	0.023	0.013	0	0.005	0	0.029*	0.035	0.043	0.074*	0.092**	0.138**	**0.092**	**0.19**	0.064**
–	–	–	–	–	–	0	0.009	0	0	0	0	0	0	0.011	0.036*	0.012	0.071**	0
–	–	–	–	–	–	–	0	0	0	0	0	0	0	0.004	0.033	0.006	0.07*	0
–	–	–	–	–	–	–	–	0	0	0.015	0.019	0.025	0.052*	0.07**	**0.11**	0.069**	**0.16**	0.043*
–	–	–	–	–	–	–	–	–	0	0	0	0	0.017	0.03*	0.062*	0.031*	**0.1042**	0.011
–	–	–	–	–	–	–	–	–	–	0.004	0.007	0.01	0.033	0.05*	0.085**	0.049*	**0.131**	0.025
0	0	0.03*	–	0	–	–	–	–	–	–	0	0	0	0.008	0.032*	0.009	**0.065**	0
0	0	0.045	–	0	–	–	–	–	–	0	–	0	0	0.004	0.026*	0.005	0.057**	0
0	0	0.012	–	0	–	–	–	–	–	0	0	–	0	0	0.009	0	0.038	0
0.006	0.001	0	–	0.011	–	–	–	–	–	0.021*	0.034	0.007	–	0	0	0	0.019	0
0.006	0.002	0	–	0.008	–	–	–	–	–	0.016	0.0262*	0.006	0	–	0	0	0.013	0
0.03	0.02	0	–	0.033*	–	–	–	–	–	0.047	0.065**	0.028*	0	0	–	0	0	0.004
0	0	0.02	–	0	–	–	–	–	–	0	0	0	0.012	0.01	0.035*	–	0.016*	0
0	0	0.003	–	0	–	–	–	–	–	0	0.004	0	0	0	0.014	0	–	0.027*
0	0	0.035	–	0	–	–	–	–	–	0	0	0	0.027	0.02	0.053*	0	0.001	–

In bold: significant values after FDR correction for multiple tests (Benjamini and Hochberg 1995). Bold values without asterisk have *P *≤* *0,001.

*P ≤* *0.05; **P ≤* *0.01.

### AMOVAs among regions

The differentiation between the three groups of samples (1: north + east coasts, 2: Gulf, 3: south + west coasts) was significant at *Glu-5′* (*F*_CT_: 0.0773; *P* < 0.000001), but neither at locus *mac-1* (*F*_CT_: −0.0003; *P* = 0.394), nor at *COI*. (*F*_CT_: −0.01244*, P* = 0.67).

### Within-region differentiation

Genetic structure was also evidenced within the three groups of samples genetically differentiated at *Glu-5′* (North, Gulf, South). In the northern group, sample AJ was significantly differentiated to all other northern samples (due to its higher frequency of allele *G* at *Glu-5′*). In this group, the sample RdA was also differentiated from all northern samples (except I3B) and had the highest frequency of allele *E* of the whole data set. In the Gulf group, the sample Ar1 was differentiated from all samples of the Gulf except BoCentre, Bo100am, and IGn. Consequently, a significant differentiation was highlighted between samples separated by no more than 500 m: Ar1 and Ar2. At locus *mac-1*, similarly, samples PR1 and PR2 appeared differentiated but only before FDR correction (Fig.1, Table4).

At the scale of the archipelago, no correlation was detected between genetic differentiation and genetic distance, at any locus. Except along the north coast, at locus *Glu-5′*, samples PCh to RdA (from northwest to east) (*P* ≤ 0.04).

### Confidence intervals of differentiations and power analyses

Jost's D values were computed for a set of seven populations from the North, Gulf, and the South regions, for *Glu-5′, mac-1* and nine allozymes. The maximum value (D = 0.049) was obtained at *Glu-5′*, and its confidence interval only overlapped that of the enzyme *PGD* (D = 0.032) which also appeared particularly differentiated (Table2)*. F*_ST_ values were all lower than those at *Glu-5′*. Additionally, whereas Jost's D confidence intervals of all loci except *Glu-5′* and *PGD* included 0.000, the *P*-values of the exact tests of overall differentiation were generally significant or highly significant (Table S2).

For *mac-1* and *COI*, we used POWSIM to simulate three populations with sample sizes and global allele frequencies corresponding to the three regions for these markers with an *F*_ST_ of 0.07 because *Glu-5*′ displayed an overall *F*_ST_ of 0.067 in Kerguelen and a *F*_CT_ of 0.077 in the AMOVA with regional groups. This value of *F*_ST_ was obtained by simulating a fission of three populations of Ne = 1000 each, that occurred 145 generations ago, parameters which allowed maintaining the observed polymorphism. Sample sizes corresponded to samples sizes for *mac-1* and *COI* in each region: 181, 725, 195 and 27, 19, 37, respectively. The allele frequencies used were 0.919 and 0.081 for *mac-1*, and 0.012, 0.012, 0.012, 0.012, 0.012, 0.012, 0.012, 0.012, 0.012, 0.012, 0.100, 0.144, 0.170, 0.060, 0.130, and 0.276, for *COI* simulations.

POWSIM simulations indicated that an overall *F*_ST_ of 0.07 would generate significant differences in more than 99.5% of the cases for the *COI* data set and in more than 92.9% of the cases for the *mac-1* data set. When there is polymorphism within populations the maximum value of fixation indices such as *F*_ST_ does not reach one even when no allele is shared among populations (Jost 2008; Meirmans and Hedrick 2011). *Glu-5′* and *mac-1* both have only two alleles, thus when comparing only two populations, *F*_ST_ values can in theory reach one and the level of intrapopulation diversity should not affect the range of possible *F*_ST_ values. For *COI,* however, the maximum possible value of *F*_ST_ was higher than at *Glu-5′*, thus a given *F*_ST_ value corresponds to less differentiation in *Glu-*5′ than in *COI* (Jost 2008; Meirmans and Hedrick 2011). Thus, the value of 99.5% of significant *F*_ST_ given by POWSIM for *COI* is an overestimate.

### Statistical analyses with environmental variables

For each environmental variable, the genetic differentiation between samples grouped by category was assessed by pairwise *F*_ST_ for each nuclear marker (Table5). None was significant at *mac-1,* but many were significant at *Glu-5′*. For the variable “Substrate”, samples collected on rocks, blocks, gravels, or sand were not significantly differentiated between each other. Regarding “Slope”, only samples collected on flat shores and hangovers were differentiated (*P* ≤ 0.0001) but steep shore samples appeared significantly differentiated neither from flat nor from hangover locations. For the three remaining factors “*Macrocystis*”, “Wave exposure” and “Salinity” the samples grouped by category were highly differentiated (*P* ≤ 0.0001). Concerning the variable “Region”, the single western sample was differentiated from northern samples only (*P* ≤ 0.004).

**Table 5:** Values of *F*_ST_ (Weir and Cockerham 1984), *F*_CT_ (AMOVA) calculated between samples of Kerguelen blue mussels grouped by categories for each environmental variable at the three loci considering either the whole archipelago or the Gulf of Morbihan. Bold values are significant after FDR correction for multiple tests per column

Variable	Locus					
	*Glu-5′*		*mac-1*		*COI*	
	Archipelago (35 pop.)	Gulf (22 pop.)	Archipelago (29 pop.)	Gulf (16 pop.)	Archipelago (23 pop.)	Gulf (12 pop.)
Wave	***F***_**ST**_**: 0.0108**	***F***_**ST**_**: 0.1315**	*F*_ST_: NS	*F*_ST_: NS	–	–
Exposure	*F*_CT_: 0.0064	*F*_CT_: 0.0123	*F*_CT_: 0.0007	*F*_CT_: −0.0023	*F*_CT_: −0.0036	*F*_CT_: −0.005
*Macro-cystis*	***F***_**ST**_**: 0.0223**	***F***_**ST**_**: 0.0096**	*F*_ST_: NS	*F*_ST_: NS	–	–
	***F***_**CT**_**: 0.0239**	*F*_CT_: 0.0125	*F*_CT_: −0.0013	*F*_CT_: −0.0025	*F*_CT_: −0.0196	*F*_CT_: −0.0168
Salinity	***F***_**ST**_**: 0.0124**	*F*_ST_: 0.0039	*F*_ST_: NS	*F*_ST_: NS	–	–
	*F*_CT_: 0.0092	*F*_CT_: 0.0033	*F*_CT_: −0.0007	*F*_CT_: −0.0023	*F*_CT_: 0.0272	*F*_CT_: 0.0135
Slope	*F*_ST_: NS	*F*_ST_: NS	*F*_ST_: NS	*F*_ST_: NS	–	–
	*F*_CT_: 0.0107	*F*_CT_: 0.0075	*F*_CT_: −0.0021	*F*_CT_: −0.0048	*F*_CT_: −0.0222	*F*_CT_: −0.0114
Substrate	*F*_ST_: NS	*F*_ST_: NS	*F*_ST_: NS	*F*_ST_: NS	–	–
	*F*_CT_: −0.0103	*F*_CT_: −0.0025	*F*_CT_: 0.0009	*F*_CT_: 0.0168	*F*_CT_: −0.0349	*F*_CT_: −0.0148

At *Glu-5′*, AMOVAs realized at the scale of the archipelago for each environmental variable, grouping the 35 samples by categories (Table5) revealed a significant differentiation between the presence and absence of *Macrocystis* (*F*_CT_: 0.0239, *P* = 0.00684). The groupings were not significantly differentiated, neither for the other factors (at *Glu-5′*), nor at *mac-1* and *COI* loci. As we tested five environmental variables, the *P*-values should be corrected taking into account multiple tests. The effect of “*Macrocystis”* presence remains significant after correction for multiple tests.

Environmental variables “Substrate”, “Wave exposure”, “Slope”, Salinity” and “*Macrocystis”* were significantly correlated to one another, except the pair “Substrate/*Macrocystis*”. The frequency of allele *G* was correlated to “*Macrocystis*” and “Region” only, whereas the variable “Region” was correlated to none of the other variables (Table6).

**Table 6:** Correlation among environmental variables and frequency of allele *G* at locus *Glu-5′* in Kerguelen blue mussels: Spearman's *ρ* values and levels of significance

Variable	Variable					
	Substrate	Slope	Wave exposure	Salinity	*Macrocystis*	Region
*G*	0.0629	0.3130	−0.2487	0.2075	0.4036*	0.3785*
Substrate		−0.6829***	0.5281**	−0.4322*	−0.2192	0.2233
Slope			−0.7176***	0.4929**	0.5210**	0.0206
Wave exposure				−0.4012*	−0.5617**	0.1474
Salinity					0.6305***	0.1025
*Macrocystis*						−0.0708

*P ≤* *0.05; **P ≤* *0.01; ***P ≤* *0.001.

AMOVAs by environmental variables, restricted to the 22 Gulf samples revealed a significant effect of “Wave Exposure” (accounting for 1.23% of the molecular variance between groups; *P *=* *0.0088) and also of the presence of “*Macrocystis”* (*F*_CT_ = 0.0125, *P *=* *0.0489), but they did not overcome the FDR correction for five tests. The presence of *Macrocystis* in a population was correlated with wave exposure in the Gulf, as most populations where *Macrocystis* occur, are exposed to waves (except PR2 which is sheltered). The effect of slope was nearly significant (*P *=* *0.058 ± 0.007). At locus *mac-1*, samples from the Gulf were differentiated (*F*_CT_ = 0.0168, *P* = 0.0289) only when grouped by “Substrate” category but this significance level did not pass the FDR correction. In the regions North (eight samples) and South (four samples), the environmental groupings did not reveal any significant differentiation at *Glu-5′* or *mac-1*. At locus *COI*, none of the environmental grouping of samples was significant.

## Discussion

### Polymorphism at the three loci and possible departures from neutral expectations

The locus *Glu-5′* has traditionally been considered as diagnostic between smooth-shell *Mytilus* species in the Northern Hemisphere (Inoue et al. 1995; Rawson et al. 1996; Borsa et al. 1999; Daguin and Borsa 2000; Daguin et al. 2001; Luttikhuizen et al. 2002; Gilg and Hilbish 2003a,b; Hilbish et al. 2003), although low frequencies of heterospecific alleles have been reported (Hamer et al. 2012). In Kerguelen, *Glu-5′* is polymorphic for heterospecific alleles and at Hardy–Weinberg equilibrium, which was unexpected in a genetic context other than the *M. edulis/M. galloprovincialis* hybrid zone in the Northern Hemisphere (Borsa et al. 2007). Mitochondria of Kerguelen blue mussels belong to the S1 clade which is endemic to the Southern Ocean (Gérard et al. 2008). The Kerguelen archipelago thus shelters the only wild and stable population (*i.e.,* outside a hybrid zone) of *Mytilus* known so far, whose polymorphism at *Glu-5′* is not in linkage disequilibrium with any of the typical genomes of northern *M. edulis*, *M. galloprovincialis*, or *M. trossulus*.

Unexpected genetic structure was here revealed at *Glu-5′* not only at the scale of the archipelago, but also at a much smaller geographic scale, down to a few hundred meters. There was a clear break in allelic frequency at *Glu-5′* between samples from the Gulf and the north coast. The highest frequencies of the allele *G* occurred in the western part of the Gulf, far from the influence of outer marine waters, and reached 60% near Mayes Island (Fig.1) (Daguin 2000; Borsa et al. 2007).

Some of *COI* haplotypes in Kerguelen blue mussels also occur in southern South America (Gérard et al. 2008). Here, we confirm the homogeneity of *COI* haplotype frequencies across the four regions of the archipelago. The shape of the haplotype network is compatible with a stable effective size of Kerguelen blue mussel population and with selective neutrality at this locus.

To summarize, in Kerguelen the polymorphism at *Glu-5′* is higher than everywhere else, whereas the polymorphism at all other nuclear loci tested (*mac-1*, *EFbis* and *EFprem's*) is lower (Bierne et al. 2002b; this study). The haplotype diversity at the mitochondrial locus *COI* is also lower in Kerguelen than in Patagonia (Gérard et al. 2008), and allozyme loci are also less polymorphic in Kerguelen than in Northern-Hemisphere populations of *M. edulis* (Blot et al. 1988). The smaller size of the Kerguelen metapopulation, compared to other less isolated populations worldwide, may explain its lower polymorphism (except at locus *Glu-5′*). Local adaptation appears as a plausible cause for the maintenance of alleles at balanced frequencies at *Glu-5′* in the heterogeneous environment of the Kerguelen archipelago.

### The three markers revealed distinct patterns of differentiation between samples

The level of differentiation is much higher at *Glu-5′* than at *mac-1*, *COI* and eight allozyme loci out of nine. One can hypothesize that allele differences at locus *mac-1* may have escaped detection because of the low resolution of agarose gels and that the power to detect possible differences at locus *CO1* may have been hampered by insufficient sample sizes. However, these hypotheses were ruled out by analyses of Jost's D and their confidence intervals, as well as the POWSIM analyses suggesting that *Glu-5′* was subjected to different constraints.

Thus, *Glu-5′* actually reveals highly significant genetic differentiation at all levels, among and within-region, and between environments. Three possible explanations arise: (i) the power analyses might be unreliable, because POWSIM uses a model of fission which may not well represent the actual situation (but Jost's D and confidence intervals are not subject to such doubts); (ii) larvae may preferentially settle (by habitat choice) in certain environments according to their genotype at *Glu-5′* or other physically linked loci; (iii) mortality or fecundity may vary among locations according to genotype at *Glu-5′* or physically linked genes (*i.e.,* differential selection). Marine species may be subjected to high variance of reproductive success (Hedgecock's sweepstake reproduction hypothesis) which together with collective dispersal of related individuals can generate complex patterns of genetic structure known as chaotic genetic patchiness (Broquet et al. 2013). A skewed offspring distribution also generates departure from the standard Kingman's coalescent and an increased heterogeneity in differentiation levels (Eldon and Wakeley 2009).

*Glu-5′* therefore seems to be an outlier displaying particularly high genetic differentiation among Kerguelen populations. However, this observation alone is not sufficient to support hypotheses of natural selection. We will thus use an additional prediction that is not well explained by purely neutral processes which is an association between genetic differentiation and environmental distance (Coop et al. 2010).

### Geographic pattern of genetic differentiation associated to ocean circulation

Patterns of genetic differentiation among Kerguelen blue mussels from different groups (North + East, South + West, and Gulf of Morbihan) similar to those here revealed at locus *Glu-5′* have been previously reported at allozyme loci (Blot et al. 1989).

After the FDR correction for multiple tests, the most significant differentiations were observed between the north coast and the Gulf. Indeed, the frontier between these regions displays the strongest break of allelic frequencies at *Glu-5′*, located between samples RdA and PAF. This sample RdA is also differentiated from all others at *mac-1*, suggesting restricted gene flow toward the most eastern point of Kerguelen. As did Blot et al. (1989), we relate the restricted gene flow to the hydrology and water masses circulation around the archipelago (Murail et al. 1977). All samples are located in the ‘Coastal Hydrological Region’ which has the most changing physical parameters even at fine scale and globally a lower salinity compared to offshore oceanic waters. However, at wider scale, the south coast and northern point of the archipelago receive the same water mass coming from the west (the ACC), but they remain isolated, thus driving to a genetic differentiation among samples from these two regions. The water masses flowing along the north and south coasts only mix far offshore in the northeastern wake zone of the archipelago (Murail et al. 1977). The presence of eddies retaining larvae on the shelf and then dragging them from a site to another on relatively short distance may explain the pattern of isolation by distance observed along the north coast at *Glu-5′*. Koubbi et al. (2000) have suggested that *Lepidonothoten squamifrons* larvae are retained by a costal gyre in the *Golfe des Baleiniers* (the open area off *Port Couvreux* (PCx), north coast) and also noted the lability of this gyre and the consequent mixing of coastal and oceanic waters during the winter period when winds are the strongest (Razouls et al. 1996; Koubbi et al. 2000). Thus, at the inter-regional scale, hydrological characteristics are able to account for the main genetic differentiation observed, by their effect on migration (i.e., without necessity to invoke selection).

### Very fine scale differentiation does not support selective neutrality

In the Gulf, a particular enclosure, genetic differentiations at very fine scale were observed: between samples from the Armor locality, Ar1 and Ar2, which are separated by very short distances (500 m) considering the dispersal potential attributed to the *Mytilus* mussels. No such differentiation is observed at the locus *mac-1*. In Armor (Ar), a marked difference in habitat occurs between samples (1 and 2). Ar1 is located near an important freshwater source, where *Macrocystis* are lacking, and has higher frequency in allele *G* than its neighbor Ar2. Out of the Gulf, in FPN a comparable habitat (Fjord with freshwater source), we also observed the same trend: a higher frequency of allele *G* compared to other south coast samples (see Table3). This trend suggests the influence of these protected, low-salinity, sandy habitat on the blue mussels that is expressed by a higher frequency of allele *G*. However, a third sample with comparable habitat shows the opposite trend: RdA has the lowest frequency of allele *G* of the whole data set (9%; see Table3).

### Genetic differentiation caused by selective pressure from environment?

At *Glu-5′*, at the scale of the archipelago, the differentiation between groups and between categories of samples with and without *Macrocystis,* were significant. Typical habitats of protected areas with flat sandy bottoms and low-salinity waters, which are more frequent in the Gulf of Morbihan, lack *Macrocystis* kelp beds. Conversely, the open coasts are mostly exposed rocky shores, bordered by *Macrocystis* beds. Consequently, searching for differentiation between samples from the Gulf and those from the south and north coasts, leads to searching the differentiation between samples located in habitats, respectively, without and with *Macrocystis* kelp beds. Finally, the genetic differentiation among the three main geographic regions may mask the environmental effect (or the reciprocal) on the genetic data. Then, analyzing environmental effect within group would avoid the ‘regions’ effect. At the within-group scale, the results were distinct, mainly due to the contrasting samplings. More precisely, the absence of significant effect of all environmental factors on *Glu-5′* data in the north and south Coasts may be due to the low number of samples (8 and 5, respectively) and/or a lower power of *Glu-5′* in these regions compared to the Gulf. In the Gulf, the *H*_n.b._ is the highest and both alleles have similar frequencies, thus allowing better detection of small differences. Indeed, within the Gulf, the substantial effect of presence/absence of *Macrocystis* beds on the sample differentiation was recovered, and the effect of the wave exposure was also revealed (see AMOVAs results). A significant result after the FDR correction cannot be considered an artifact of the number of AMOVAs that were carried out. The environmental effects found by the AMOVAs (even within the Gulf of Morbihan) do not necessarily reflect habitat choice or differential selection linked to *Glu-5′* genotypes: geographically close populations tend to share environmental characteristics even within region (for instance, the numerous samples from the *Henri Bossière* Fjord are all similar) thus if there is fine scale structure due to any other factor, by indirect correlation, a statistical effect of environment may arise even in the absence of causal relationship.

To conclude, three independent lines of evidence suggest that *Glu*-5′ is affected by selection (or habitat choice): (i) the high polymorphism at this locus in Kerguelen, (ii) highest and more significant *F*_ST_ and *F*_CT_ at *Glu-5′* compared to other loci, and (iii) the significant effects of environmental factors on AMOVAs even within region. However, none is a sufficient proof of selection by itself.

## Supporting Information

**Table S1.**Molecular markers: locus name, source, primer sequence, annealing temperature (T°C) and fragment length (L) in base pairs. **Table S2.** Kerguelen blue mussels.
