# Supplementary material for: Does natural selection explain the fine scale genetic structure at the nuclear exon Glu-5′ in blue mussels from Kerguelen?
Source: Ecol Evol. 2015 Mar 6;5(7):1456–73. doi: 10.1002/ece3.1421 (PMC4395175; doi:10.1002/ece3.1421)
Supplement: Supplementary file 1 — Table S1.Molecular markers: locus name, source, primer sequence, annealing temperature (T°C) and fragment length (L) in base pairs. Table S2. Kerguelen blue mussels. [file ece30005-1456-sd1.docx]

# ***Supplementary material to:*** *Does natural selection explain the fine scale genetic structure at the nuclear exon Glu-5' in blue mussels from Kerguelen?*

*by K. GERARD, C. ROBY, N. BIERNE, P. BORSA, J.-P. FERAL, and A. CHENUIL*

**Table S1.** Molecular markers: locus name, source, primer sequence, annealing temperature (T°C) and fragment length (L) in base pairs

| Locus | Primer | Sequence | T°C | L | Reference |
| --- | --- | --- | --- | --- | --- |
| *Glu-5'* | *Me15* | CCAGTATACAAACCTGTGAAGAC | 54°C | 210/160 | Inoue et al. (1995) |
|  | *Me17* | CTGGTGGATAATTTGTCT TTGC |  |  | Daguin (2000) |
| *mac-1* | *Mac1ex1-F* | GCTGTATTTCCATCAATTGTTGG | 58°C | 370/470 | Daguin & Borsa (1999) |
|  | *Macmyt-R* | CGAAAATTGTAGTCTAGTTTTGTG |  |  |  |
| *EFbis* | *EFbis-F* | ACAAGATGGACAATACCGAACCACC | 52°C | 400 | Bierne et al. (2002) |
|  | *EFbis-R* | CTCAATCATGTTGTCTCCATGCC |  |  |  |
| *EFprem's* | *EFprem's-F* | TTCATCAAGAACATGATCACTGG | 54°C | 400 | N.B. unpublished |
|  | *EFprem's-R* | CACAGCACAATCAGCTTGAGATG |  |  |  |
| *COI* | *LCO1490* | GGTCAACAAATCATAAAGATATTGG | 59 | 645 | Folmer *et al.* (1994) |
|  | *AM-HCO* | TAAACYTCAGGGTGMCCAAAAAAYCA |  |  | Gérard et al. (2008) |

**Table S2.** Kerguelen blue mussels. Average H_S_: within population average expected heterozygosity; H_T_: total expected heterozygosity as estimated from electromorph frequency data at 9 allozyme loci (Blot 1989; Blot *et al.* 1989). Nine population samples (54-80 individuals, all but one ‘locus x population combination’ having more than 71 individuals genotyped) were genotyped from a similar regional sampling as ours. NA: allele numbers; F_ST_: computed F_ST_ based on allele frequency data using F_ST_ = 1- (Average H_S_ / H_T_); P: P-values obtained from an exact test based on contingency tables of allele numbers per population and testing the null hypothesis that allele frequencies are similar among populations, using the nine population sample; P (North): same as P among the three North populations; P (South): same as P among the four Gulf populations.

| Locus | Average H_S_ | H_T_ | NA | F_ST_ | P | P (North) | P (Gulf) |
| --- | --- | --- | --- | --- | --- | --- | --- |
| *LAP1* | 0.644 | 0.651 | 5 | 0.010 | **0.000** | **0.000** | 0.078 |
| *LAP2* | 0.405 | 0.417 | 3 | 0.027 | **0.000** | 0.063 | **0.000** |
| *PGD* | 0.317 | 0.334 | 3 | 0.052 | **0.000** | **0.001** | **0.000** |
| *PGI* | 0.642 | 0.649 | 4 | 0.012 | **0.002** | 0.884 | **0.003** |
| *PGM* | 0.402 | 0.412 | 4 | 0.023 | **0.000** | **0.019** | 0.341 |
| *GOT1* | 0.016 | 0.015 | 2 | -0.005 | 0.175 | ns | ns |
| *GOT2* | 0.040 | 0.040 | 2 | 0.018 | **0.001** | 0.265 | **0.000** |
| *MDH1* | 0.078 | 0.079 | 2 | 0.009 | 0.005 | 0.606 | 0.335 |
| *MDH2* | 0.041 | 0.042 | 2 | 0.023 | **0.000** | 0.366 | **0.036** |
